# Retrieval-induced forgetting of spatial position depends on access to multiple shared features within categories

**DOI:** 10.3758/s13421-022-01384-1

**Published:** 2023-01-09

**Authors:** Shaohang Liu, Josie Briscoe, Christopher Kent

**Affiliations:** School of Psychological Science, University of Bristol, 12a, Priory Road, Clifton, Bristol, BS8 1TU UK

**Keywords:** Spatial cognition, Interference/inhibition in memory retrieval, Memory, Recall

## Abstract

Retrieval-induced forgetting (RIF) is typically observed in verbal memory tasks, although a few studies have observed RIF in visual spatial tasks. This leaves an open question as to whether RIF depends on semantic identity to link across semantic properties of objects, or whether RIF depends on access to the perceptual features of objects. To explore RIF of spatial positions, we report three experiments utilizing a continuous measure of the accessibility and precision for objects that were distinguished by their shape, color, and spatial region. After a study phase, half of the objects in a single-color category were selectively practiced for their spatial position, by requiring the object to be placed in the exact spatial position seen previously. Finally, all objects were probed for their spatial position at test. No RIF occurred for objects that shared only one color feature but were located within the same spatial region (in Experiment 1) or when objects shared the same color, but were located within different spatial regions (in Experiment 3). However, RIF did occur when objects shared the same spatial region and the same color (Experiment 2). Overall, the interim recall of the spatial positions of cue-objects impairs access to the position of other cue-objects within the same color category, but only when these groups had sufficient overlapping and competing features. The finding that RIF only occurs to the accessibility of spatial positions, not the precision of visual spatial memory, was interpreted as consistent with inhibitory theories of forgetting.

## Introduction

The experimental exploration of forgetting has been ongoing for well over a century with a consensus that forgetting can be caused by the interaction of new memoranda with existing memories (e.g., Postman, 1971). The inhibition theory of forgetting (Anderson, 2003) attributes forgetting to cognitive control processes (e.g., inhibition) that reduce the interference arising from the auto-activation of existing memories that compete with retrieval of the target. This view is partly motivated by the experimental observation that retrieving from long-term memory can cause the temporary unavailability of items in the same semantic category; an effect referred to as *retrieval-induced forgetting* (i.e., RIF; see Anderson, 2003, for a review).

The standard RIF paradigm was first reported by Anderson et al. (1994) and consists of three phases. Initially, in a study phase, participants try to memorize multiple exemplars taken from several categories (e.g., FRUIT-APPLE, FRUIT-BANANA, FRUIT-ORANGE and ANIMAL-CAT, ANIMAL-DOG, ANIMAL-HAMSTER). Then, participants selectively practice a subset of the exemplars from a subset of the categories (e.g., FRUIT-APPLE). This manipulation generates three distinct groups of items: practiced exemplars in the practiced categories (Rp+ items; FRUIT-APPLE), unpracticed exemplars in the practiced categories (Rp- items; FRUIT-BANANA), and unpracticed exemplars in the unpracticed categories (baseline items, BL; ANIMAL-DOG). Finally, participants are asked to recall the items that were shown in the initial study phase by their semantic identity (we refer to the object label, e.g., APPLE, as its semantic identity, to distinguish this from its perceptual form, e.g., a spherical green object). Typically, retrieval practice of the semantic identity of a selected few objects increases recall of Rp+ items compared to BL items (akin to the testing effect). Importantly, forgetting is observed when the unpracticed Rp- items are recalled less frequently than BL items, even though neither were practiced. According to an inhibition account, the occurrence of RIF is due to the inhibition of Rp- items. These Rp- items generate relatively more interference and competition compared with BL items when trying to retrieve Rp+ items (e.g., BANANA competes with APPLE, whereas DOG does not).

Retrieval-induced forgetting has typically been investigated using verbal materials such as word-pairs (e.g., Anderson et al., 1994) and facts (e.g., Campbell & Thompson, 2012), but has rarely been examined using non-verbal materials (e.g., visual objects). In one exception to this, Ciranni and Shimamura (1999) had participants memorize the spatial position of different shapes categorized by distinguishable colors. The experiments revealed that recalling the spatial position of the shapes impaired the memory for shapes within the same color category. Another study by Gómez-Ariza et al. (2012) also reported a spatial RIF by asking participants to memorize the position of words. When the word target was cued with either its color or with semantic identity (by providing two letters), then RIF occurred for the spatial position of the word. These investigations indicate a potentially important aspect of RIF: RIF can occur for both semantic and episodic memories where episodic memory reflects perceptual and contextual attributes of the stored memory. Intriguingly, Tempel and Frings (2014) used novel words with meaningless category labels (e.g., FEX- HINDUGOTT), but still obtained a significant RIF. As the authors state, simply sharing a context (in this case an arbitrary category label) can create a relationship between items sharing this context sufficient to cause RIF.

A benefit of using visual stimuli over verbal stimuli is the possibility of obtaining a continuous measure of memory performance. Rather than obtaining a discrete recalled/not recalled response to each item, participants can be asked to recall a feature that varies on a continuous dimension such as color (e.g., Zhang & Luck, 2008). This enables exploration of an important issue in visual-spatial RIF studies: are Rp- items inaccessible (i.e., they temporarily cannot be retrieved) or are the representations of Rp- items less precise? Previous studies investigating RIF for the spatial position of targets have used discrete measurement of recall accuracy (correct/incorrect) that does not differentiate between a lack of accessibility and the loss of memory precision. In Gómez-Ariza et al. (2012), the screen was split into segments and the target appeared in a fixed position within a segment. In this case, participants could have encoded the position information with a coarse semantic label (e.g., “top-left”) rather than maintaining a fine-grained visual memory for the exact position. If so, successful retrieval of targets could reflect the use of a verbal-labelling strategy, not the integrity of spatial memory. Similarly, in Ciranni and Shimamura (1999), the shapes were located around 12 segments of a circle. It is easy for participants to consider the circle as a clock and then use the “time” (e.g., 9 o’clock) to encode and recall the position. That is, in these examples, a coarse semantic label can result in a correct response in the final recall phase but potentially mask a lack of precision of the visual memory representation. Therefore, a discrete measure of retrieval performance does not differentiate between two possible sources of failure; either retrieving the memory fails because an item becomes inaccessible, or because the precision of the item representation becomes degraded in memory.

The present study employed a novel RIF paradigm in which memory performance for the visual-spatial position of an item was measured as a continuous variable enabling memory precision and accessibility to be estimated. Our methods are based on Zhang and Luck (2008), who introduced a paradigm for visual working memory where participants were asked to recall the color of objects using a continuous color wheel. Responses were assumed to reflect a mixture of memory for color retrieved from the stored representation, and guessing due to the inability to access the representation of the target object’s color. If the response was guessed, it should fall within a uniform distribution of responses that have an equal probability of occurrence at any point on the color wheel (i.e., a circular uniform distribution). If the response was veridical to the color target, it should fall within a von Mises distribution (as equivalent to a circular normal distribution) where (a) there is a maximum probability of making a response on the color wheel at the point of an exact match to the correct color hue, and (b) the probability of responding gradually decreases as color hue shifts further from the target. Using a mixture-modelling approach, the proportion of responses falling within a uniform distribution and a von Mises distribution was estimated to represent the proportion of guessed responses and veridical responses, respectively. The dispersion parameter for the von Mises distribution indicates the precision of veridical responses i.e., the wider the dispersion, the lesser the memory precision. Following previous research (e.g., Liu, 2022; Zhang & Luck, 2008), the present study adapted a similar continuous measure of memory for visual-spatial position. Participants were asked to retrieve visual-spatial position by dragging an object to its original position within a specified display as accurately as possible. Responses were coded as spatial co-ordinates of the final position of a target. Using mixture-modelling, a guessing distribution that was drawn from the likelihood of random responding within a specified area was segregated from a veridical response distribution to estimate the accessibility and precision of memory for target positions, respectively.

In another study in our laboratory (Liu, 2022), we used a color-wheel methodology similar to Zhang and Luck’s (2008) method to analyze a non-verbal variant of RIF with object color as the memory target. Participants memorized the *arbitrary* color of an exemplar shape (e.g., pink RABBIT) from different categories (e.g., ANIMAL). During retrieval practice, participants selectively recalled the color of a subset of exemplars that were sampled from numerous categories (four categories for each block; six blocks in total). Using a continuous color wheel, the recalled target color feature was indicated by clicking on the closest match on the wheel. In the final test, participants recalled the color of all exemplars using the color wheel. Across five experiments, a robust observation of forgetting of the color feature was demonstrated such that retrieving the color feature of the target (e.g., a pink RABBIT) impaired recall of color features of Rp- exemplars from the same semantic category (e.g., a blue DOG). That is, even though the memory targets (the color feature) were not competing at a featural level, RIF occurred. Mixture-modelling confirmed that forgetting only impaired the accessibility of color features from memory and not the precision of the Rp- items. In the study by Liu et al., *semantic* competition was diminished by (a) using color features as memory targets and (b) assigning arbitrary object-color associations. However, their design still relied on conceptually rich objects (e.g., RABBIT, DOG) that corresponded to explicit semantic categories (e.g., ANIMALS). In the present design, semantic competition was diminished further by (a) using visual-spatial position as the target feature, (b) relying on simple geometric shapes with arbitrary object-color associations, and (c) using simple color features to define categories. This provides a more rigorous test of whether RIF occurs within a semantically impoverished design.

The present study implemented the procedure of a typical RIF paradigm with visuo-spatial stimuli instead of (more typical) verbal stimuli. Each target item constituted a distinct geometric shape in a unique position and the shapes were organised into two arbitrary color categories. Responses to the exact target position were practiced for a subset of items within a single color category (Rp+ items). Therefore, the memory target comprised the co-ordinates of the spatial position of the target, and not its semantic label or any distinguishing features of target identity. Typically, in verbal RIF tasks, the association between target semantic identity and its category generates competition that is thought to elicit inhibition of Rp- items. Here, the association is semantically impoverished and so a decrease in inhibition of the Rp- items would result in a diminished RIF, if lexico-semantic activation were the driving factor of RIF. As RIF persists within non-verbal paradigms, we anticipate that retrieving the spatial position of the targets will impair the spatial memory for competitors in the same category. Using the mixture-modelling approach we can determine whether RIF in spatial memory could be linked to a failure to access memory representations, or to less precise visual-spatial memories.

## Experiment 1

### Method

#### Participants

Forty-eight undergraduate psychology students from the University of Bristol (30 females and 18 males; M_age_ = 20.3 years, SD = 1.98) were recruited for course credit. Full consent was obtained from all participants and ethical approval for the study was obtained from the appropriate University Research Ethics Committee.

#### Design and materials

The learning materials were 16 unique simple geometric shapes (e.g., a square, cross, rectangle) that were presented in five blocks of trials. Within each block, half (eight) of the shapes were presented in the same color (e.g., green) and the remaining shapes were filled with a second color (e.g., red). Each block contained two types of items that were presented with a unique color-position pairing. Across blocks, shapes were segregated into five color categories (red-green, blue-yellow, black-white, pink-purple, and orange-indigo). Each block consisted of (a) a study phase, in which all 16 items were shown; (b) a retrieval practice phase, in which half of one color category were tested (RP+ items), and (c) a final test phase, in which all the study items were tested. The experiment was programmed using Psychopy 1.90.3 (Peirce, 2007) and was displayed on a 60-cm diagonal screen. Participants sat approximately 30 cm away from the screen. All stimuli were displayed within a grey circle with a diameter of 400 pixels, centered at the middle of the screen.

#### Procedure

**Fig. 1:**
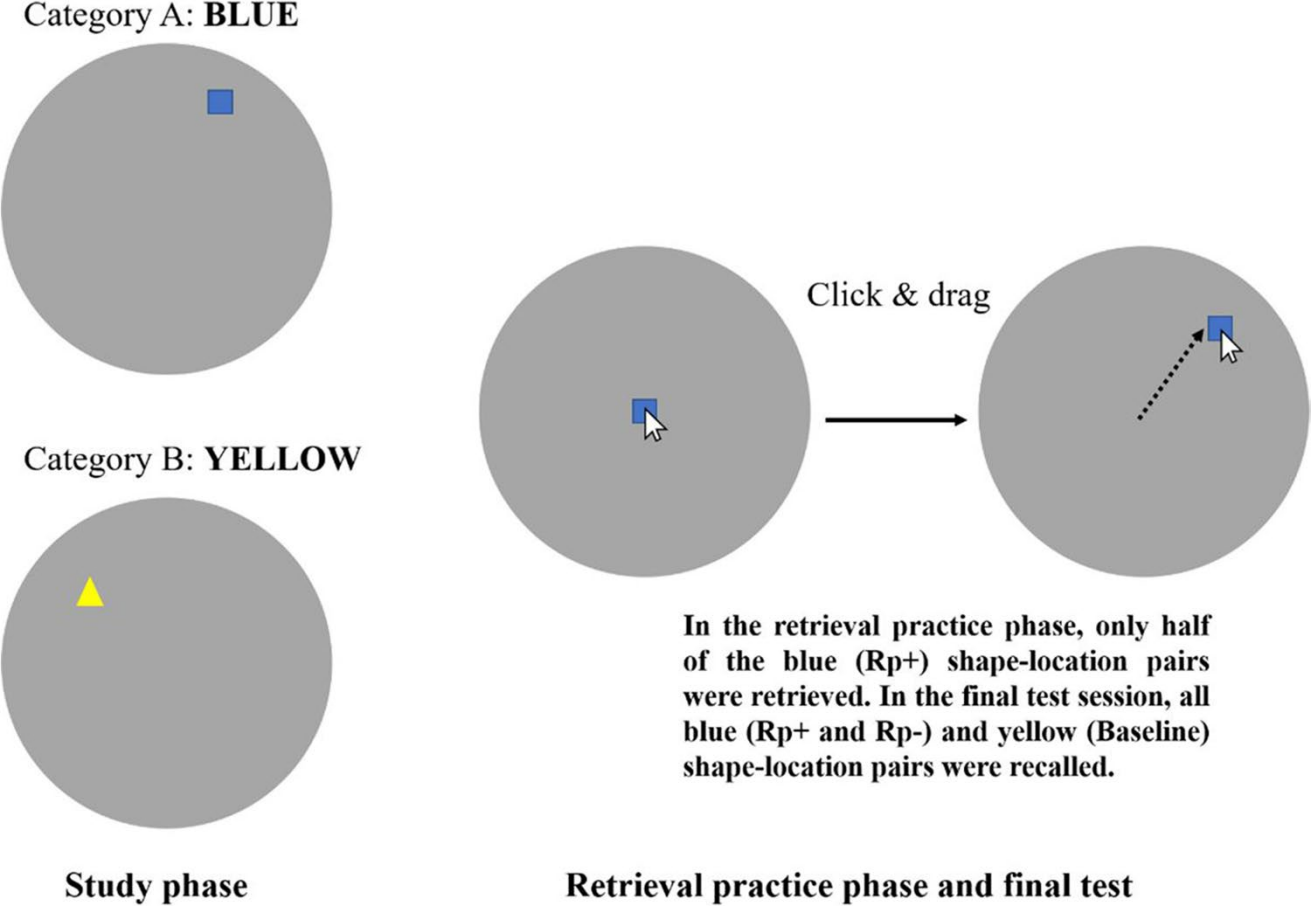
The position of shapes from two color categories were learned in the study phase, but only half of the items with one color were retrieved in the practice phase (Rp+ items). Participants recalled the shape position by clicking and dragging the object from the middle of the circle. All shape-position pairings were tested in the final test

The present study followed the standard RIF paradigm, as shown in Fig. 1. In the study phase, each shape was displayed at a randomly^1^ selected position outside the central 1/20th of the circle to generate a distinguishable position (i.e., the possible stimulus field was an annulus). Each shape was displayed for 5 s with a 500-ms inter-stimulus interval. For the retrieval practice phase, four items from one color group were randomly selected as to-be-practiced items (RP+). In the practice phase, shapes were presented in the middle of the circle and participants needed to drag the shape to the correct position that corresponded to the position observed in the study phase. Participants used the left button of the mouse to drag the shape and the right button to proceed to the next trial. Each shape was presented twice for retrieval practice. Then, a distractor task required participants to spend 1 min on a semantic fluency task where they needed to input as many exemplars as possible according to a given category (in this experiment and subsequent experiments country names were used as the category cue). Finally, in the test phase, participants were asked to recall the position of all 16 shapes, presented in a random order, following the same procedure as the retrieval practice phase. The procedure was replicated for five blocks where each block used a different pair of color categories.

#### Data analysis

The analysis of variance and post hoc tests were computed using JASP (JASP Team, 2022). Data analysis was conducted in two stages. First, we used the absolute cartesian distance between the response position and correct position as a direct measure of participants’ spatial memory. Specifically, the distance was represented by the distance between the coordinates of stimulus position and response position. Second, we used a mixture-modelling approach (similar to Zhang & Luck, 2008) to partition the distribution of absolute response error (ARE) into two distributions representing guessed (random) responses and genuine retrieval attempts based on items that are considered as “successfully recalled” items by the model.

One issue with using the mixture modelling approach is how to determine what the distribution of guessing responses should be. In previous investigations, the guessing distribution was typically represented by assuming a (circular) uniform distribution, for example when the probability of selecting any single point on a circular color wheel is equiprobable (Zhang & Luck, 2008), or when the probability of selecting the direction of heading of a moving target (Crowe et al., 2019), or with a uniform distribution when selecting a point within a two-dimensional space (Crowe et al., 2019). In the present study, the shape positions were generated randomly so we can assume the guessing distribution derives from the distances (*d*) between two random points (P_0_ and P_1_) within an annulus (i.e., a circle that omits the center where the items did not emerge in the study phase). For this reason, assuming a uniform distribution would be inappropriate as the probability for different *d* values occurring is not equiprobable. For example, it is almost impossible to obtain a *d* close to the length of diameter because this will only occur when the original and the response point are reaching the end points of a diameter.

**Fig. 2:**
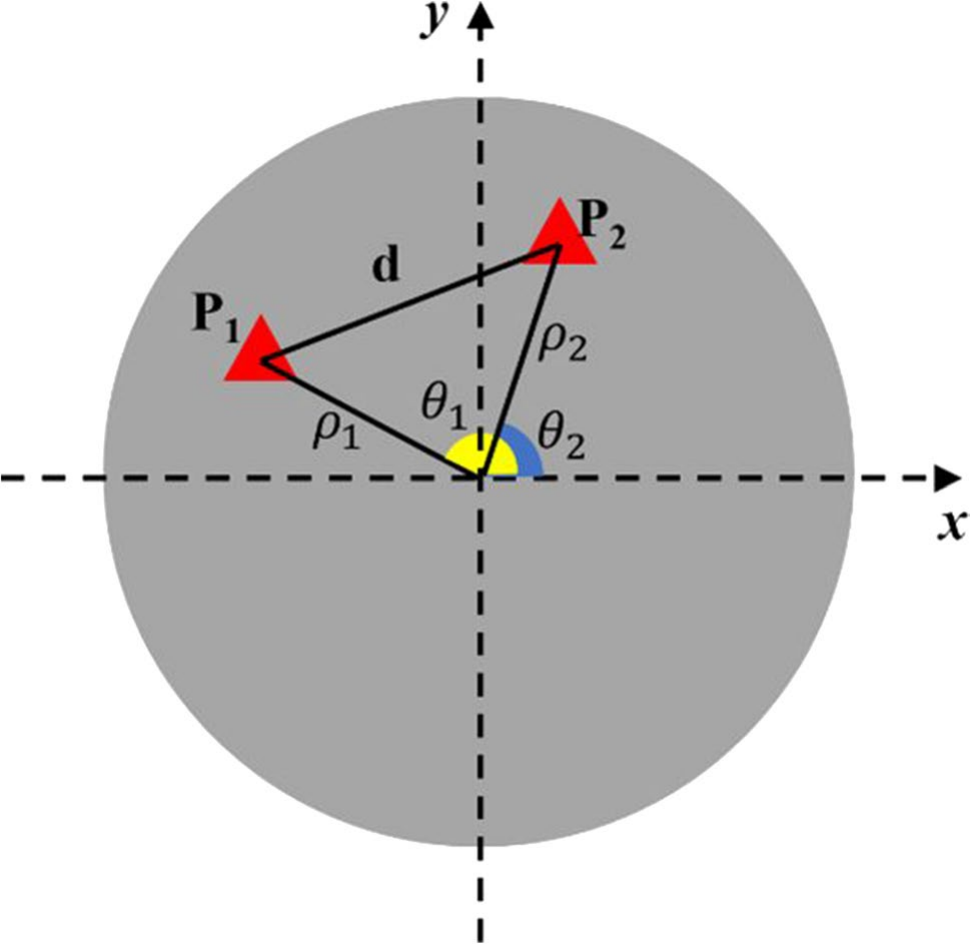
The original and response points (P_1_ and P_2_) were represented by polar coordinates (polar angle θ and polar axis ρ). *d* is the distance between point P_1_(ρ_1_, θ_1_) and response point P_2_(ρ_2_, θ_2_). The domain of ρ_1_ and ρ_2_ is (0.05R, R); the domain of θ_1_ and θ_2_ is (0, 2π)

**Fig. 3:**
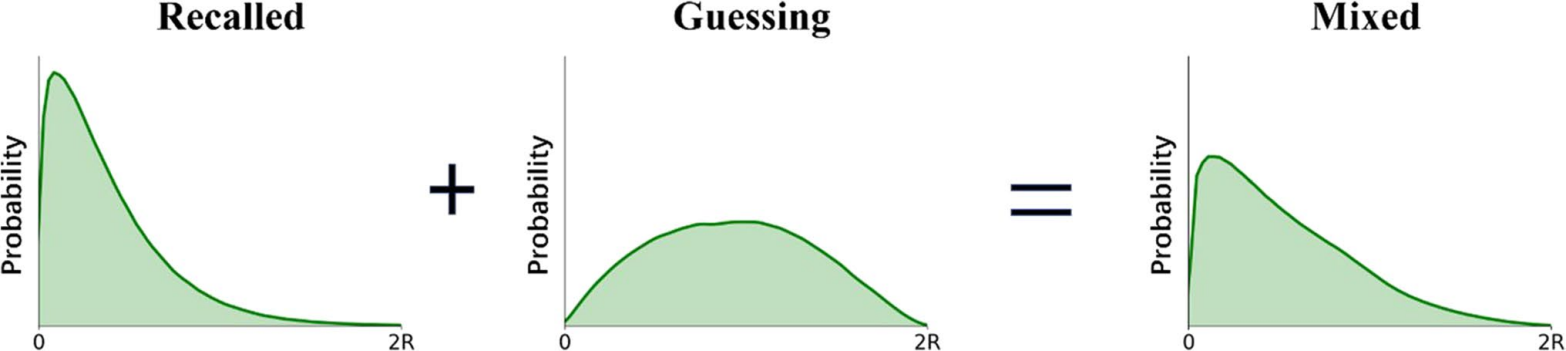
Participants’ responses consist of guessing responses and responses based on retrieved information about the position of shapes (which is noisy). A guessing distribution was determined from the distribution of two-point distances occurring at random within the response annulus. A Weibull distribution was used to represent the distribution of retrieved information responses where the scale parameter corresponded to the memory precision of retrieved positions. The proportion of responses under the Weibull distribution represents the proportion of recall-based responses, and the remaining proportion represents guessing responses. The scale parameter of the Weibull determines the precision of participants’ memory representation (small values of the scale is associated with greater memory precision)

In order to estimate the distribution of guessing, we used polar coordinates to indicate points within an annulus, where a point is defined by two parameters of polar axis (ρ) and a polar angle (θ). The distance *d* between the original point P_1_(ρ_1_, θ_1_) and response point P_2_(ρ_2_, θ_2_) can be calculated by the following formula (see Fig. 2):

1 $$d=\sqrt{{\rho_1}^2+{\rho_2}^2-2{\rho}_1{\rho}_2\cos \left({\theta}_1-{\theta}_2\right)}$$

where the maximum value of ρ is the radius and minimum value is the radius of the central part (0.05R). The range of θ is from 0 (0°) to 2π (360°). Therefore, ρ1 and ρ2 can be drawn from a uniform distribution from 0.05R to R; θ1 and θ2 can be drawn from a uniform distribution from 0 to 2π. A guessing distribution of *d* can therefore be generated (see Fig. 3). Additional analysis of participants’ absolute responses showed that the assumption that participants responded randomly was reasonable, and participants made full use of the available space (for details of this analysis, see OSF link: https://osf.io/3d7bk/?view_only=2ae23330b6324eeaa2b349e6f8fddacc).

To describe the distribution of recalled items, a Weibull distribution was used where both the shape parameter and the scale parameter can be varied to approximate other distributions, including the normal distribution (see also Crowe et al., 2019). Here, the Weibull scale parameter was used to indicate the precision of the memory representation, as the scale parameter controls the spread of the distribution. Figure 3 shows how the mixture model is generated. Three parameters were estimated – the proportion of responses captured by a Weibull distribution (i.e., successfully recalled items), the scale (representing the precision of the representation), and the shape parameters of the Weibull distribution. The parameters were obtained by a Bayesian method using Monte Carlo and Markov Chain (MCMC) modelling with 10,000 iterations. The mean and 95% confidence interval (CI) of highest density posterior was used as the estimated value for each parameter. Mixture modelling analysis was accomplished by using Pymc3 (a Python-based library for probabilistic programming; see Patil et al., 2010).

### Results

**Fig. 4:**
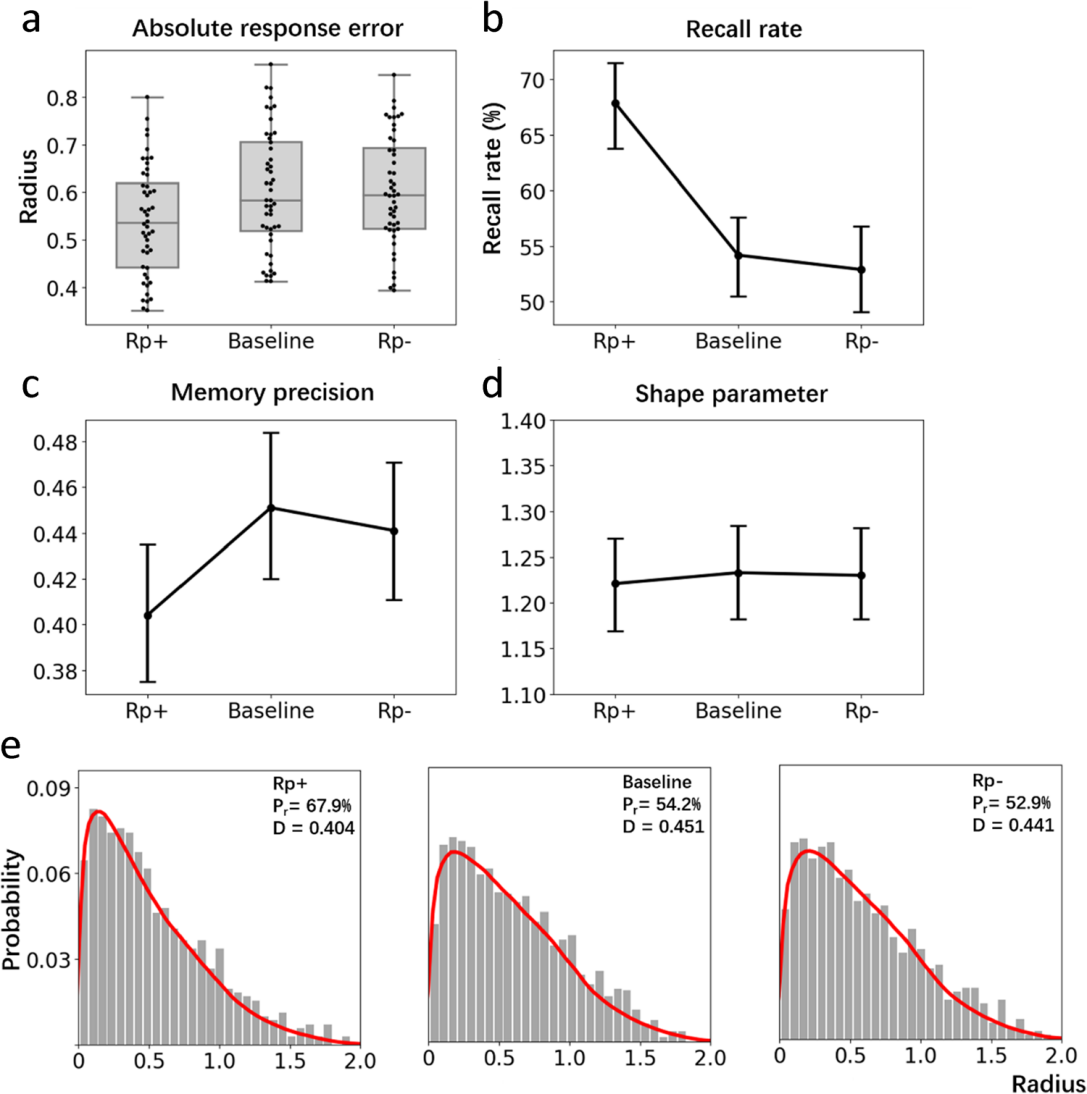
**a** Box plots show ARE distributions for the three stimulus types. Interquartile range is indicated by the size of boxes and the black line in the boxes represents the median of each distribution. Dark points indicate the individual data points. **b** Estimated recall rates (as a proportion of Weibull distribution) for each condition. Error bars indicate 95% confidence intervals in Panels b, c and d. **c** Estimated memory precision (from the scale parameter of the Weibull distribution, higher scores represent greater variance and hence lower precision). **d** Estimates of the shape parameter of the Weibull distribution. **e** Histograms show the distribution of pooled data for each condition where the red curve is a simulated mixture distribution based on the estimated parameters, namely the distribution of guessed items plus the distribution of recalled items (Weibull distribution)

Figure 4a displays distributions of ARE for the three item types (Rp+, Rp-, and BL). An initial check was performed to ensure performance within these conditions was above chance level. We estimated chance performance by randomly generating 100,000 distances within the annulus of the experimental display. The mean of these random distances was 0.925 radians. A one-sample t-test revealed that all three conditions were all above chance level (Rp+: *t*(47)= 23.06, *p* < .001, *d* = 3.33, *BF*_*10*_ = 9.86e+29; *BL*: *t*(47)= 17.66, *p* < .001, *d* = 2.55, *BF*_*10*_ = 5.46e+22; Rp-: *t*(47) = 19.16, *p* < .001, *d* = 2.77, *BF*_*10*_ = 1.97e+23). Comparing performance across item types, a three-way repeated-measures ANOVA revealed a reliable difference in ARE, *F*(2, 94) = 10.47, *p* < .001, *η*^*2*^_*p*_ *=* .182, *BF*_*01*_ = 287.62. Using Bonferroni’s correction, post hoc tests only detected a significant difference between Rp+ and BL, *t*(47) = 3.95, *p* < .001, *d* =.571, *BF*_*01*_ = 107.90; Rp+ and Rp-, *t*(47) = 3.97, *p* < .001, *d* = .573, *BF*_*01*_ = 491.41. No difference between Rp- and BL, *t*(47) = 0.02, *p* = 1.00, *d* = 0.003, *BF*_*01*_ = 0.092 was observed. So, while a testing effect emerged (RP+ > BL), there was no RIF effect (BL > RP-).

As no previous studies have measured RIF by absolute distance (instead relying on proportion recalled), we conducted a sensitivity analysis (using G-power 3.1.9.7, Faul et al., 2007) to ensure there was sufficient power to detect effects. With power set to .80 we could detect an effect as small as *η*^*2*^_*p*_ = .033 for a three-way repeated-measures ANOVA.

Parameter estimates were produced by pooling data points from all 48 participants (720 data points from Rp+ and Rp- conditions and 1,440 data points from BL condition). Means and 95% CIs of estimated parameters were generated. We identified statistically significant differences between two parameters by judging whether the mean of one parameter is out of the range of the 95% CI of the other parameter, even though the 95% CI of two parameters can sometimes overlap slightly. Figure 4b shows the estimated recall rate as estimated from the fitted Weibull distribution for the three conditions. Participants recalled significantly more Rp+ items (67.9%, *CI:* +3.9%, -4.1%) than BL (54.2%, *CI*: +3.8%, -4.0%) and Rp- items (52.9%, *CI*: +4.1%, -4.1%). An identical pattern was obtained for memory precision (i.e., the scale parameter of the fitted Weibull distribution) where Rp+ items (0.404, *CI*: +0.029, -0.030) were more precisely recalled than BL (0.451, *CI*: +0.028, -0.031) and Rp- items (0.441, *CI*: +0.029, -0.031). As is shown in Fig. 4d, and as expected, there was no difference in the shape parameter of the fitted Weibull distributions across conditions (Rp+: 1.230, *CI*: +0.052, -0.049; BL: 1.221, *CI*: +0.051, -0.051; Rp-: 1.240, *CI*: +0.048, -0.052).

### Discussion

After studying color-shape pairs and their respective visual-spatial position, participants were able to retrieve shape positions at above-chance levels. A retrieval practice benefit was observed for shape-position pairs, where the opportunity of retrieval practice increased both the memory precision and accessibility of Rp+ items. Retrieval-induced forgetting effects did not emerge in Experiment 1 for the Rp- items, relative to the unpracticed BL items. Although performance for Rp- items was above chance, there was no difference in the relative accessibility or precision of memory for these items. Despite the correspondence between the color categories shared between the Rp+ and Rp- items, there was no evidence of more forgetting of the Rp- items. This finding implies that sharing one visual feature (such as color) across a set of items is not sufficient to generate RIF for arbitrarily paired features (such as shape and position) of single items. It is possible that the color features did not provide a sufficient cue to the category information necessary to instigate relative forgetting of items within the same category. To further understand the boundaries of RIF with visuo-spatial material, we sought to investigate the number of overlapping features that are necessary to observe RIF. To do so, we manipulated the relative strength of the cues to their category membership in Experiment 2; this was increased in order to generate stronger discrimination between items from *different* categories and stronger *within* category competition. If category assignment underpins forgetting in the RIF paradigm, then increasing the strength of within-category competition should increase the likelihood of observing RIF with visual-spatial stimuli. Therefore, in Experiment 2, members of the same category shared two features: color and spatial region (top or bottom half within the annulus).

## Experiment 2

### Method

#### Participants

Forty-five undergraduate psychology students (25 males and 30 females; M_age_ = 20.9 years, *SD* =1.12) were recruited from the University of Bristol for Experiment 2 and participated in return for course credit. Full consent was obtained for all participants and ethical approval for the study was obtained from the appropriate University Research Ethics Committee. A sensitivity analysis identical to Experiment 1 showed we could detect an effect size as small as *η*^*2*^_*p*_ = .036.

#### Design and materials

**Fig. 5:**
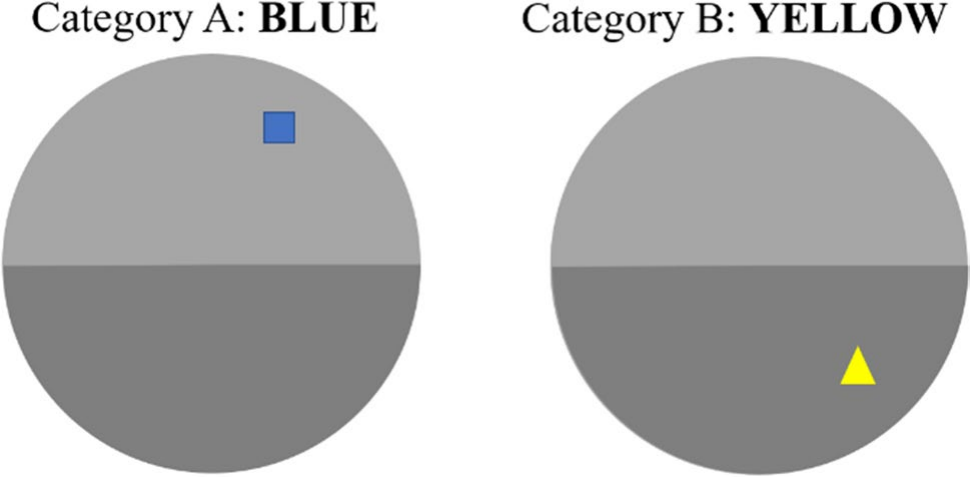
For Experiment 2, a critical manipulation was introduced that segregated categories by pairing the color with the semi-annular region of the item: Each shape only emerged with one color and in one semi-annulus in the study phase

The design of Experiment 2 was identical to Experiment 1, except for one critical difference. As displayed in Fig. 5, only one category (color) of items was associated with positions occurring in one half of the annulus. Specifically, shapes of the same color could only occur within the same semi-annulus (top or bottom half of the annulus) within the study phase so that both the region and color provided clues to category membership. In other respects, the materials and procedure were identical to Experiment 1, apart from one procedural constraint. In the retrieval practice and testing session, participants were not permitted to drag a shape to the opposing semi-annulus where the color and semi-annulus pairing did not correspond, i.e., position responses to the item were confined to the corresponding category of the item.

#### Data analysis

**Fig. 6:**
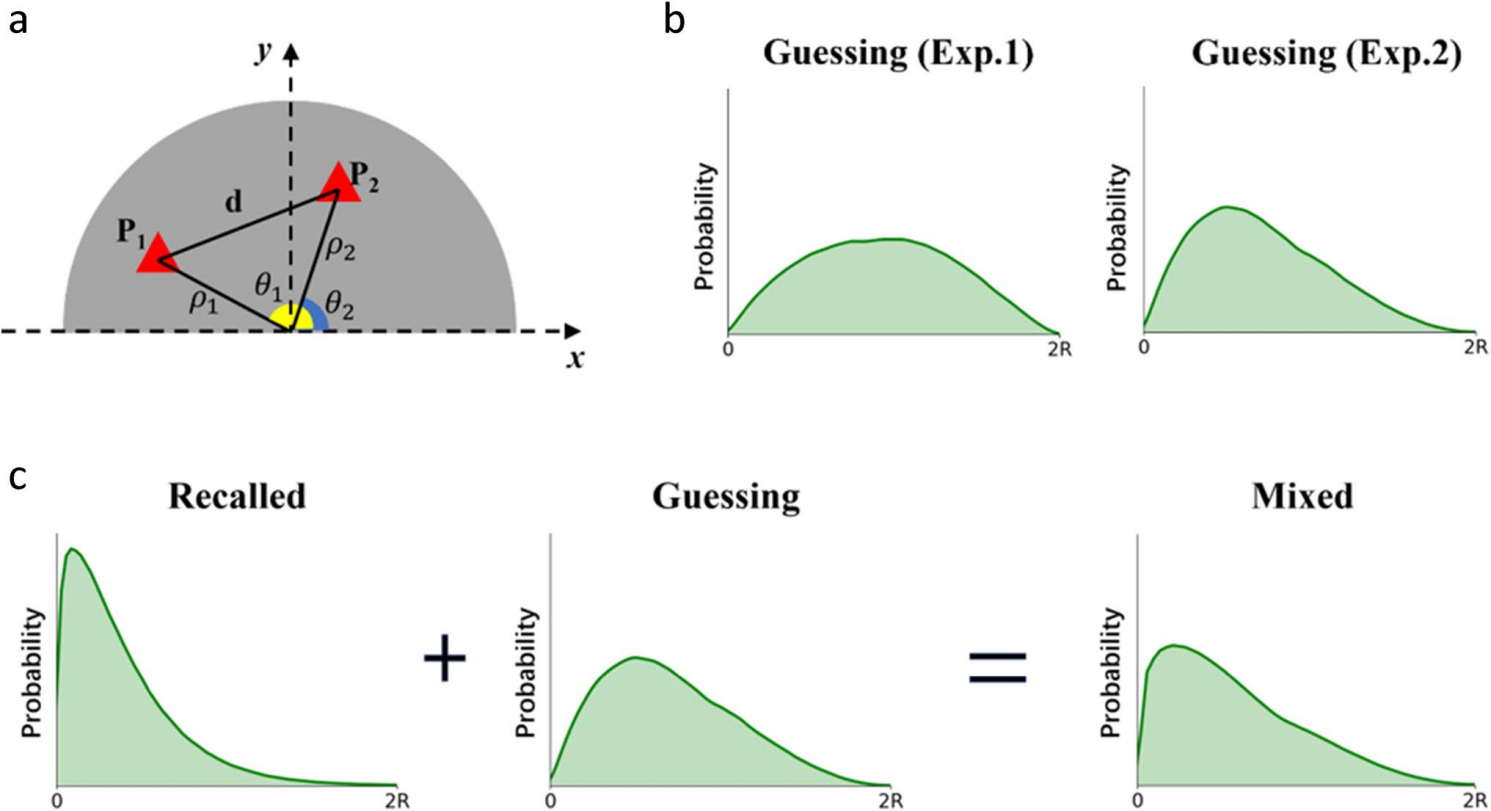
**a** The original and response points (P_1_ and P_2_) were represented by polar coordinates (polar angle θ and polar axis ρ). d is the distance between point P1(ρ1, θ1) and response point P2(ρ2, θ2). As one color category only occurred in one semi-annulus in the initial study phase, the domain of θ1 and θ2 is from 0 to π. **b** Different guessing distributions were generated due to the material difference between Experiments 1 and 2 – the response area was a semi-anulus, instead of a whole anulus in Experiment 2. This produces a guessing distribution with higher skewness. **c** Identical to Experiment 1, participants’ responses consist of guessing responses and genuine responses to the position of shapes. A guessing distribution was determined from the distribution of two-point distances occurring at random within the response semi-annulus. A Weibull distribution was used to represent the distribution of genuine positions retrieved where the scale parameter corresponded to the memory precision of retrieved positions. The proportion of responses classified to Weibull distribution represents the proportion of recall-based responses, and the rest of the proportion represents guessing responses

The data analysis for Experiment 2 followed an identical logic to the analyses conducted for Experiment 1, apart from one aspect. By constraining responses to one semi-annulus at test, it was necessary to adjust the estimates for the guessing distribution of responses within a semi-annulus. For Experiment 2, as is shown in Fig. 6a, the guessing distribution was estimated from distances between two random points within a semi-annulus (i.e., a semi-annulus that omits the central 1/20th). Thus, although the distance between original point P1(ρ1, θ1) and response point P2(ρ1, θ1)) can still be calculated according to Equation 1, the area of θ1 and θ2 should be changed from (0, 2π) to (0, π). Likewise, if the objects only emerged in the bottom semi-annulus, the domain of θ1 and θ2 should be (π, 2π). Figure 6b shows the difference between two guessing distributions estimated for different domains of θ. Figure 6c depicts the components of the distribution of response error – a guessing distribution and a recall-based distribution (Weibull distribution).

### Results

**Fig. 7:**
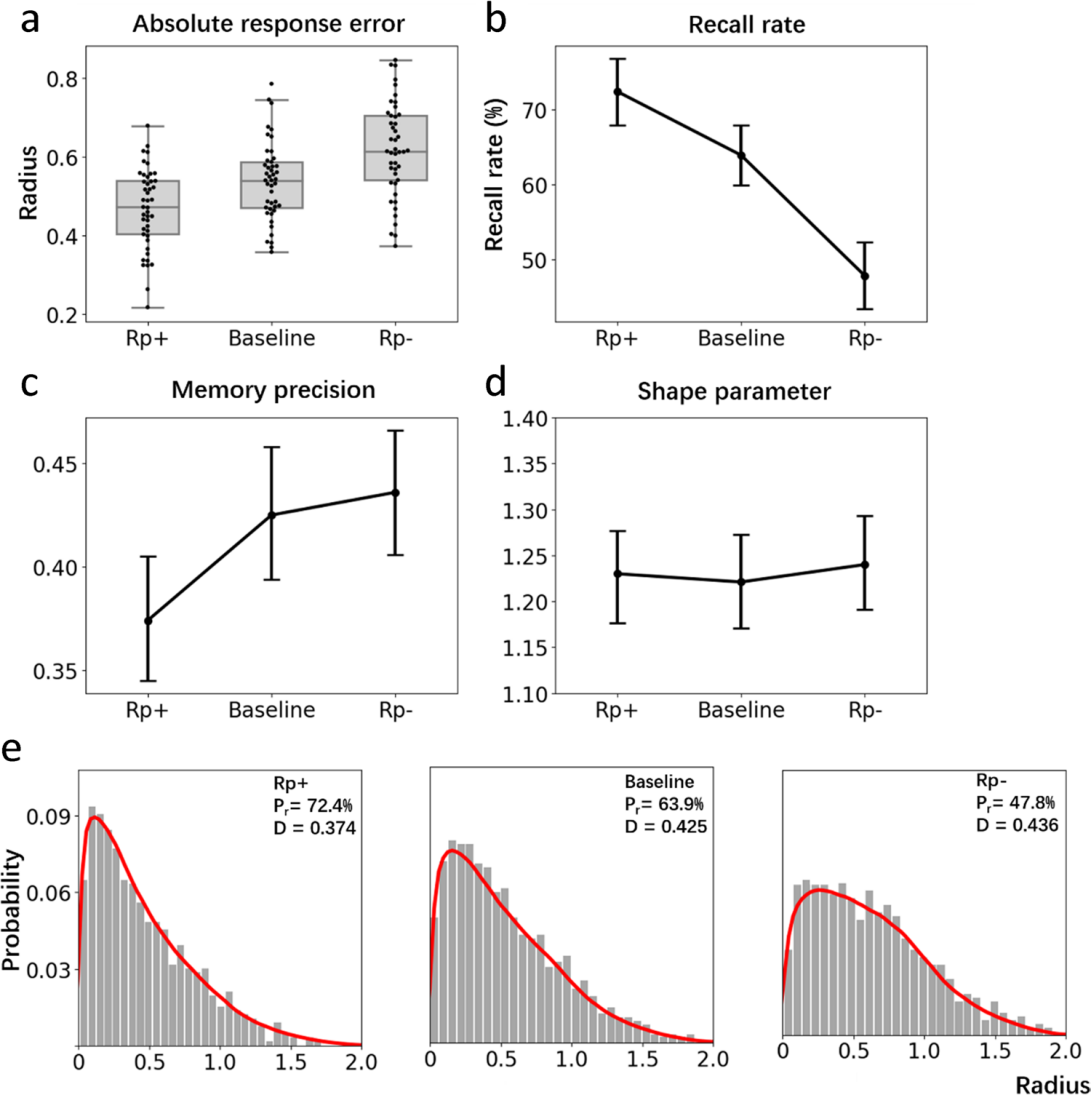
**a** Box plots show absolute response error (ARE) distributions for the three stimulus types. Interquartile range is indicated by the size of boxes and the black line in the boxes represents the median of each distribution. Dark points indicate the individual data points. **b** Estimated recall rates (as a proportion of Weibull distribution) for each condition. Error bars indicate 95% confidence intervals in Panels b, c and d. **c** Estimated memory precision (from the scale parameter of the Weibull distribution). **d** Estimates of the shape parameter of the Weibull distribution. **e** Histograms show the distribution of pooled data for each condition where the red curve is a simulated mixture distribution based on the estimated parameters, namely the distribution of guessed items plus the distribution of recalled items (Weibull distribution)

Figure 7a displays the distribution of ARE (in radians) for the three item types. Chance level responding was again estimated by sampling 100,000 random distances from a single semi-annulus. The mean of these random distances was 0.705 radians. A one-sample t-test indicated that performance on all three conditions was above chance (Rp+: *t*(54) = 15.54, p < .001, *d* = 2.32, *BF*_*10*_ = 1.24e+121; *BL*: *t*(54) = 11.28, *p* < .001, *d* = 1.68, *BF*_*10*_ = 3.94e+121; Rp-: *t*(54) = 4.78, *p* < .001, *d* = .71, *BF*_*10*_ = 1.02e+118) . To establish whether performance varied systematically across item types, a repeated-measures ANOVA was conducted that demonstrated reliable differences between conditions in ARE, *F*(2, 88) = 35.83, *p* < .001, *η*^*2*^_*p*_ *=* .449, *BF*_*01*_ = 3.54e+9. Post hoc Bonferroni-corrected t-tests indicated significantly higher ARE for the Rp- condition compared with the Rp+ condition (*t*(54) = 3.90, *p* < .001, *d* = 1.26, *BF*_*01*_ = 3.30e+8) and the BL (*t*(54) = 4.56, *p* < .001, *d* = .68, *BF*_*01*_ = 172.07. ARE was also significantly lower for the Rp+ condition compared to the BL condition (*t*(54) = 3.81, *p* < .001, *d* = 0.58, *BF*_*01*_ = 130.37).

Data points were pooled from 45 participants to obtain parameter estimates (675 data points for Rp+ and Rp- conditions, and 1,350 data points for BL condition). Means of posterior distributions were reported for each estimated parameter. As displayed in Fig. 7b, the Rp+ condition (72.4%, *CI*: +4.0%, -4.0%) achieved the highest recall rate compared to the BL condition (63.9%, *CI*: +3.9%, -3.8%) and to the Rp- (47.8%, *CI*: +3.9%, -4.0%) condition, demonstrating a robust testing effect. Critically, RIF was observed from the significantly lower recall rate for the Rp- condition compared to the BL condition.

From the mixture-modelling, only the Rp+ condition (0.374, *CI*: +0.030, 0.031) showed an advantage for memory precision (i.e., as observed from the scale parameters of the Weibull distributions) compared to the other two conditions; no difference was identified between the scale parameters for the Rp- (0.425, *CI*: +0.032, 0.030) and BL conditions (0.436, *CI*: +0.032, -0.032). It was also clear that the shape parameters of the Weibull distributions did not differ across the three conditions (Rp+: 1.224, *CI*: +0.047, -0.049; BL: 1.233, *CI*: +0.050, -0.0481; Rp-: 1.230, *CI*: +0.051, -0.049).

### Discussion

Retrieval-induced forgetting was evident in Experiment 2 when contextual information from the color (i.e. shapes within a single semi-annulus) and spatial region of shape targets co-occurred in order to distinguish two categories of shapes. The mixture-modelling of the retrieved spatial positions generated parameter estimates for retrieved items and their corresponding features. From these parameter estimates, the distributions for the Rp+ condition has a larger scale parameter that describes the spread, or stretch, of the Weibull distribution. Additionally, the Rp+ condition also contained a higher estimated contribution from the Weibull distribution than the other two conditions. This confirmed the benefit of retrieval practice in both Experiment 1 and Experiment 2 for both memory accessibility and precision of the Rp+ items. The nature of RIF was revealed by the comparison between BL and Rp- condition; RIF only impaired the accessibility of memory rather than the quality of the visual-spatial memory.

The critical manipulation of the number of shared features was important to observing either the presence (in Experiment 2) or the absence (in Experiment 1) of RIF of visual-spatial positions. The observation that the presence of RIF depends on the number of overlapping features suggests that RIF is moderated by the relative strength of category information. If more convergence of featural information generates greater coherence within categories and/or a stronger basis for category assignment, then having sufficient category strength is necessary for observing RIF. As the feature information was collated from perceptual features to form categories, the findings of Experiment 2 further demonstrate that visually categorized material can yield RIF in a similar way to verbally categorized material. That is, the observed effects are consistent with RIF reported in other domains. However, another possibility not ruled out by Experiment 2 is that the spatial region (semi-annulus position) alone, rather than the combination of color and spatial region, is sufficient to cause this visual RIF. Therefore, we conducted Experiment 3 to examine this possibility by manipulating only the spatial region cues. In Experiment 3, all the shapes were in the same color but occurred in only one semi-annulus that differed dependent on color. If Experiment 3 produces the same pattern of null effects as Experiment 1, then one could affirm that it is the number of overlapping features generated within a category that accounts for this inconsistency, rather than the type of feature that is shared across targets.

## Experiment 3

### Method

#### Participants

Fifty participants (20 males and 30 females; *M*_*age*_ = 25.9 years, *SD* =1.89) were recruited from a Chinese university for Experiment 3 and they acquired 20 yuan (about US$3) for participation. All participants received information about the study and provided full consent for participation and use of their data. *η*^*2*^_*p*_ .032.

#### Design and materials

The design of Experiment 3 was identical to Experiment 2, but all the shapes were filled with an identical black color hue such that color was not a cue to category membership.

#### Data analysis

The data analysis for Experiment 3 followed an identical logic to the analyses conducted for Experiment 2.

### Results

**Fig. 8:**
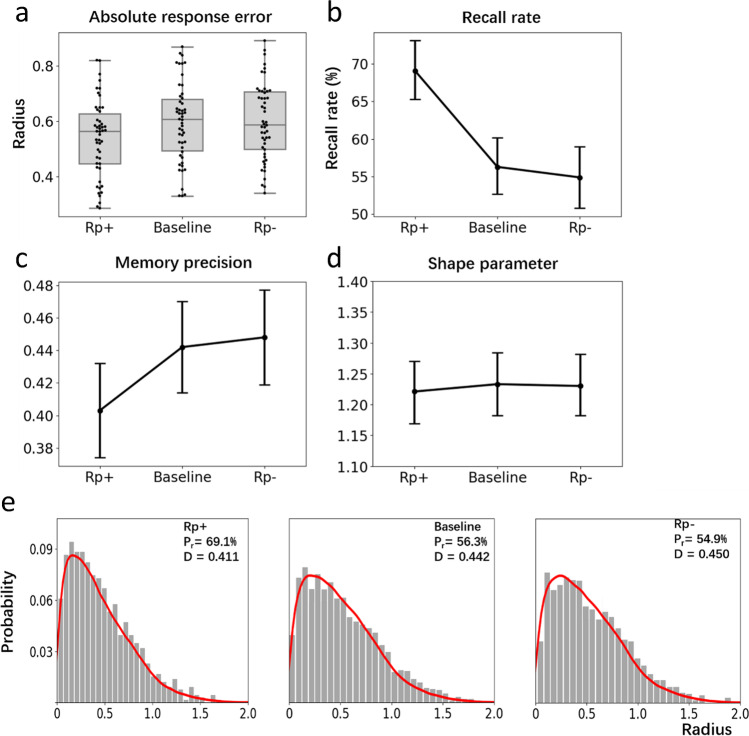
**a** Box plots show absolute response error (ARE) distributions for the three stimulus types. Interquartile range is indicated by the size of boxes and the black line in the boxes represents the median of each distribution. Dark points indicate the individual data points. **b** Estimated recall rates (as a proportion of Weibull distribution) for each condition. Error bars indicate 95% confidence intervals in Panels b, c and d. **c** Estimated memory precision (from the scale parameter of the Weibull distribution). **d** Estimates of the shape parameter of the Weibull distribution. **e** Histograms show the distribution of pooled data for each condition where the red curve is a simulated mixture distribution based on the estimated parameters, namely the distribution of guessed items plus the distribution of recalled items (Weibull distribution)

Figure 8a displays distributions of ARE for the three item types (Rp+, Rp-, and BL). An initial check was performed to ensure performance within these conditions was above a chance level of responding. As for Experiment 2, random guessing was estimated as 0.705 radians. A one-sample t-test revealed that all three conditions were all above chance level (Rp+: *t*(49)= 5.37, *p* < .001, *d* = 0.76, *BF*_*10*_ = 2.76+e8; *BL*: *t*(49) = 5.40, *p* < .001, *d* = 0.76, *BF*_*10*_ = 8831; Rp-: *t*(49) = 16.47, *p* < .001, *d* = 2.77, *BF*_*10*_ =8194). Comparing performance across item types, a three-way repeated-measures ANOVA revealed a reliable difference in ARE, *F*(2, 98) = 5.23, *p* = .006, *η*^*2*^_*p*_ *=* .096, *BF*_*01*_ = 5.02. Using a Bonferroni correction, post hoc tests only detected a significant difference between Rp+ and BL, *t*(49) = 2.70, *p* = .024, *d* =.41, *BF*_*01*_ = 3.39; Rp+ and Rp-, *t*(49) = 2.89, *p* < .001, *d* = .38, *BF*_*01*_ = 8.02. No difference between Rp- and BL, *t*(49) = 0.20, *p* = 1.00, *d* = 0.03, *BF*_*01*_ = 0.15 was observed. So, while a testing effect emerged (RP+ > BL), there was no RIF (BL > RP-).

Parameter estimations were produced by pooling data points from all 50 participants (750 data points from Rp+ and Rp- conditions and 1,500 data points from BL condition). Means and 95% CIs of estimated parameters were generated. Figure 8b shows the estimated recall rate (i.e., estimated proportion from the Weibull distribution) for the three conditions. Participants recalled significantly more Rp+ items (69.1%, *CI*: +3.8%, -4.0%) than BL (56.3%, *CI*: +3.6%, -3.9%) and Rp- items (54.9%, *CI*: +4.1%, -4.1%). An identical pattern was obtained for memory precision (i.e., the scale parameter of the fitted Weibull distribution) where Rp+ items (0.411, *CI*: +0.029, -0.030) were more precisely recalled than BL (0.442, *CI*: +0.028, -0.031) and Rp- items (0.450, *CI*: +0.029, -0.031). As is shown in Fig. 8d, and as expected, there was no difference in the shape parameter of the Weibull distributions across conditions (Rp+: 1.249, *CI*: +0.050, -0.048; BL: 1.244, *CI*: +0.046, -0.044; Rp-: 1.235, *CI*: +0.047, -0.047).

### Discussion

Experiment 3 replicated the same qualitative pattern of findings observed in Experiment 1, despite testing a different feature of the objects i.e., spatial region rather than color. This supported our explanation for the absence of RIF in Experiment 1, i.e., that the occurrence of RIF requires a sufficient number of features to overlap to generate a strong enough within-category similarity between category members (as seen in Experiment 2). Additionally, the results of mixture modelling support the findings of Experiments 1 and 2 in that the retrieval benefits (for Rp+ items) increased both the accessibility and precision of the memory representations.

## General discussion

We demonstrated that RIF could occur in a non-verbal paradigm when the spatial position of simple shapes must be remembered, i.e., when category information is available only from visual-spatial features and when the memory targets correspond to visual-spatial features. This result is consistent with a previous investigation that also tested RIF with non-verbal materials (Ciranni & Shimamura, 1999) in that RIF can occur when the lexical competition between Rp+ and Rp- items is minimized and when these items share a perceptual feature(s). Also, RIF can occur with overlap between (peripheral) perceptual features, even when the semantic identity (such as shape and color) has been provided but not retrieved. With a more rigorous implementation of the RIF paradigm that provided continuous measurement of memory performance, these experiments provide further evidence that RIF only impairs the memory accessibility of the competitors, rather than the precision of the retrieved memory.

In the present study, the identity of the memory target (e.g., the colored shape) was provided at study and test, but only the featural information pertaining to spatial position was actively retrieved. So, activating memory for target position and any overlapping features that relate across items did not necessarily require the central identity of the object to be actively retrieved. These findings can be contrasted with those of Gómez-Ariza et al. (2012) in which recalling the identity of an object impaired the memory of competitive objects from the same lexical semantic category and its location. Gómez-Ariza et al. (2012) showed that when retrieving multi-featured objects, their spatial position could be activated automatically by access to object identity; the feature overlap at the heart of RIF occurred whether the memoranda were shapes or words. From this, it is clear that RIF occurs across different degrees, or levels, of representation of concepts.

In the standard verbal RIF, effects can occur by mapping the associative relations between the identity of practiced (Rp+) concepts to the identity of unpracticed concepts (Rp-). RIF can also occur through other forms of mapping. First, RIF is elicited by mapping the associative relations from the semantic identity of unpracticed concepts (Rp- items) to their perceptual features (also Rp+ items; e.g., Gómez-Ariza et al., 2012). Second, RIF is elicited by mapping from one perceptual feature of Rp+ items to the same perceptual feature of Rp- items (as seen in Experiment 2), but where the semantic identity (as defined by its shape and label) is constant. It is unclear if the transfer of RIF from feature-to-feature mapping occurs for different features, even within visual spatial memoranda. For example, it would be interesting to determine whether retrieving the color features can induce the forgetting of the position of related objects.

Fundamentally, the inhibition theory of RIF (e.g., Anderson et al., 1994) claims that forgetting occurs due to processes of inhibition linked to the activation of concepts. This claim is based primarily on evidence of inhibitory processing within the activation of lexical-semantic identity (by object labels) and of related concepts (by category). In the present work, RIF did not occur in Experiments 1 and 3, but did occur in Experiment 2 when there was a relative increase in the strength of category cueing information. This increase in category strength led to stronger discrimination between objects from different categories and an increase in within-category similarity, with presumably concomitant effects on patterns of activation and competition. It was clear that by having either a single color feature (Experiment 1) or a single spatial feature (Experiment 3) as the only shared feature with other exemplars of the same category, the category relations were not sufficient to cause RIF. RIF only occurred when an additional *arbitrary* feature (i.e., the spatial region and the color of the item) was conjoined by association to the target (Experiment 2). This finding aligns with the conclusion of Tempel and Frings (2014), who observed RIF when a meaningless label comprised the category information: a lexical-semantic association of a category-exemplar relation is neither a pre-condition for RIF, nor the only source of RIF. The arbitrary association between the semantic identity and category points to patterns of activation and competition that builds around perceptual (i.e., the present study) and contextual relations (Tempel & Frings, 2014).

An interesting aspect of the current research is that, with only one feature overlapping, RIF did not occur. RIF only emerged when the Rp+ and Rp- items shared two features. However, previous studies have successfully observed RIF with only a single feature overlap (Ciranni & Shimamura, 1999). One possibility for the inconsistency with previous work is that, in the present research, participants can rarely rely on a coarse semantic label (e.g., “top-left”) to complete the task (i.e., a label that indicates the accurate position of the shape). This variation may have increased the participants’ cognitive load and so altered the threshold of competitive activation at which RIF was triggered. As inhibition theory (Anderson, 2003) states, the occurrence of RIF critically relies on an automatic inhibitory control of potential competitors, and studies have indicated that this process can be interrupted when cognitive resources are insufficient (Pica et al., 2013). According to Pica et al. (2013), when the task becomes harder, for example, from remembering the position within finite options, to indicating the accurate position of the target, more motivation and attention is needed to permit the engagement of inhibitory control, which may consequently eliminate RIF.

The inclusion of the mixture-modelling analysis in the present study allowed us to investigate where the benefits of retrieval practice and the relative impairment of Rp- items occur. In all three experiments, there was a stable benefit for the memory precision of Rp+ items, relative to unpracticed Rp- and BL items. Additionally, the guessing rate was lower for Rp+ items, indicating that Rp+ items were more accessible in memory. An insight as to the origin of RIF is provided by the difference in memory accessibility for Rp- items in Experiment 2, where RIF impaired memory accessibility but not memory precision. The notion of this accessibility-only impairment for RIF corresponds to the assumptions of an inhibition account where competitors are inhibited due to a shared category cue, but where the whole representation remains intact and retrievable at a later time, (e.g., RIF typically dissipates after 24 h; MacLeod & MacRae, 2001).

Mixture modelling is used to elucidate the pattern of guessing and informed decision making across a wide range of tasks (e.g., Crowe et al., 2019; Sutterer & Awh, 2015; Zhang & Luck, 2008). It can provide a useful insight into patterns of responding and the underlying processes alongside accuracy and response time measures. However, the interpretation of mixture modelling and its underlying assumptions is not without issue in the visual working memory literature (e.g., Luck & Vogel, 2013; Ma et al., 2014). For example, this approach assumes that random errors can be distinguished from other putative sources of response error that are difficult to interpret. We found the distribution of absolute responses was consistent with the assumption of random guessing and there was a good fit of the mixture-modelling showing that it can segregate the non-random errors. We interpret the non-random responses as generated from retrieval based on the stored item representation and the variance of the distribution as related to the precision of the representation. We note that our conclusions about the occurrence of RIF in visual spatial material can be derived directly from the ARE data without reliance on the assumptions of mixture modelling.

In conclusion, RIF within verbal memory is well established; however, fewer studies have considered RIF outside of verbal material and pre-existing semantic category structures. With a novel version of the RIF task that combined a visual spatial design with mixture modelling of continuous response data, these experiments demonstrated that RIF can map from the semantic identity of an object to its perceptual features, and also map from its perceptual features to semantic identity. In other words, lexical-semantic competition is not necessary for RIF to occur but that emerging competition triggered by sharing visual contextual information and visual features is sufficient to generate RIF. Therefore, RIF can operate across both semantic and episodic contexts, even when the perceptual features of objects are arbitrarily combined to form temporary categories. Using mixture modelling implied that memory precision is unaffected, so the inhibited memory representation can be retrieved at a later point and still retain the vividness of its perceptual qualities. Since the effects of RIF are relatively temporary, these findings reiterate the adaptive role of incidental forgetting. Memories that arise from predominantly episodic features (feature and contextual association) can be temporarily blocked to reduce interference, but bound representations are not broken up in a way that has lasting consequences for the fate of these items in memory. Understanding RIF across different contexts can inform theories of both the adaptive and maladaptive role of forgetting in everyday experience.

## Footnotes

1 After conducting the experiments we realized the position of stimuli was not truly random due to an issue with the random number generator in Python. This resulted in a slight bias to present stimuli within a square region extending -282 to 282 pixels (R/√2). However, for an individual participant, any slight non-random bias in stimuli position was highly unlikely to be detected.
